# Synergistic Effects of K_2_CO_3_ and Melamine on Enhancing the Performance of Date Stone‐Derived Supercapacitors

**DOI:** 10.1002/open.202500271

**Published:** 2025-06-16

**Authors:** Atif Saeed Alzahrani

**Affiliations:** ^1^ Materials Science and Engineering Department King Fahd University of Petroleum & Minerals Dhahran 34464 Saudi Arabia; ^2^ Interdisciplinary Research Center for Sustainable Energy Systems (IRC‐SES) King Fahd University of Petroleum & Minerals, KFUPM Dhahran 34464 Saudi Arabia

**Keywords:** date stones, energy storages, melamine, nitrogen‐doped activated carbon, supercapacitors

## Abstract

Biomass‐derived carbon materials play a crucial role in advancing electrochemical energy storage technologies toward a cleaner and more sustainable future. This study investigates the potential of date stone biomass as a sustainable precursor for fabricating high‐performance supercapacitor electrodes. A two‐stage pyrolysis process is utilized, incorporating K_2_CO_3_ as an activating agent and melamine as a nitrogen dopant. The synergistic interaction of these additives results in an activated carbon material with a significantly improved surface area, pore volume, and nitrogen content. The electrochemical analysis reveals a high specific capacitance of 209.36 F g^−1^ at 0.5 A g^−1^, excellent cycling stability over 5000 cycles at 10 A g^−1^, and high energy and power densities (24.47 Wh kg^−1^ at 2500 W kg^−1^). This study demonstrates the feasibility of utilizing date stone biomass as a renewable resource for advanced carbon‐based materials in energy storage applications, contributing to a more sustainable future.

## Graphical abstract

This study showcases date stones as a sustainable source for high‐performance supercapacitors. A two‐stage pyrolysis with potassium carbonate and melamine created a nitrogen‐doped carbon with a high surface area. The resulting electrode achieved 209.36 F g^−1^ specific capacitance, excellent cycling stability, and high energy/power densities (24.47 Wh kg^−1^ at 2500 W kg^−1^), demonstrating its promise for advanced energy storage applications.

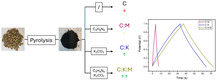

## 1 Introduction

The increasing urgency to mitigate greenhouse gas emissions has prompted many countries to commit to achieving a net‐zero carbon economy. To accomplish this, electrochemical energy storage has emerged as a key technology, facilitating the transition to a low‐carbon economy by enabling the electrification of transportation and electricity sectors—the primary sources of greenhouse gas emissions.^[^ ^1^ ^]^ Electrochemical energy storage systems primarily consist of three components: the cathode, anode, and electrolyte. Electrolytes function as electrically insulating yet ionically conductive media, effectively separating the cathode and anode electrodes while enabling ion transport. Furthermore, the cathode and anode electrodes store electric charge during the charge and discharge cycles.

Electrochemical energy storage technologies encompass a diverse range of systems, which can be classified based on their charge storage mechanisms into two primary categories. 1) Faradaic, which involves electron transfer processes and phase transformations, such as those observed in metal‐ion batteries and 2) non‐Faradaic, which stores energy through electrostatic interactions only, as seen in supercapacitors.^[^ ^2^, ^3^ ^]^

Due to differences in charge storage mechanisms, metal‐ion batteries typically exhibit high energy density, moderate power density, and good cyclability (e.g., >200 Wh kg^−^^1^, <1 kW kg^−1^, with thousands of charge cycles before failure). On the other hand, supercapacitors are recognized for their ultra‐high‐power density and exceptional cycling stability (e.g., 4–6 Wh kg^−1^, 1–2.5 kW kg^−1^, and tens of thousands of cycles before failure).^[^ ^4^, ^5^ ^]^ Supercapacitors are used in applications such as regenerative braking systems in electric vehicles, portable consumer electronics, and microgrid energy management.^[^ ^6^ ^]^ However, enhancing their energy density remains a critical challenge for broader adoption and expanded functionality.^[^ ^7^ ^]^

Enhancing the energy density of supercapacitors can be achieved by increasing the rated voltage and/or capacitance. The rated voltage is primarily determined by the choice of electrolyte, as electrolytes with a higher electrochemical stability window enable an expanded voltage range, thereby improving the overall energy storage capability.^[^ ^8^ ^]^ Conversely, capacitance is directly related to electrode surface area and the ability to facilitate surface redox reactions, a process known as the pseudocapacitive charge storage mechanism.^[^ ^6^ ^]^ Among various electrode materials, carbon‐based materials are the preferred choice due to their structural tunability and chemical versatility. Carbon materials can be synthesized with diverse dimensional structures, including 0D (quantum carbon dots), 1D (carbon nanotubes), 2D (graphene), and 3D (graphite), each offering distinct advantages for energy storage applications. Moreover, the structural integrity and porosity of these materials can be controlled, further enhancing their electrochemical performance.

In addition, heteroatom doping (e.g., nitrogen,^[^ ^8^, ^9^, ^10^ ^]^ sulfur,^[^ ^11^, ^12^ ^]^ and phosphorus^[^ ^13^, ^14^ ^]^) has been widely employed to modify the electrical and chemical properties of carbon materials. The incorporation of these nonmetal dopants alters electrical conductivity, surface wettability, and electrochemical activity, thereby improving overall capacitance. By carefully optimizing processing conditions and precursor selection, it is possible to tailor the physical and chemical properties, therefore, adjusting electrochemical properties of carbon‐based electrode materials.^[^ ^15^ ^]^

Despite their advantages, the majority of commercial carbon materials are derived from nonrenewable fossil resources, particularly petroleum‐based precursors, which pose significant environmental concerns due to their carbon footprint and extraction‐related emissions. As a result, the development of sustainable, renewable carbon sources has gained increasing attention. Biomass, an abundant and renewable organic resource, serves as a promising alternative for producing high‐performance carbon materials.^[^ ^16^ ^]^

Through pyrolysis and activation, biomass can be transformed into highly porous activated carbon materials, suitable for supercapacitor electrodes and other electrochemical applications.^[^ ^17^, ^18^ ^]^ The utilization of biomass for energy storage offers dual benefits. It mitigates environmental waste accumulation while simultaneously providing cost‐effective and high‐performance electrode materials.^[^ ^19^ ^]^ However, biomass is inherently complex, containing cellulose, hemicellulose, lignin, and various minor organic and inorganic constituents.^[^ ^20^ ^]^ Consequently, significant research efforts have been directed toward investigating novel biomass‐derived carbon materials, including those sourced from pine sawdust,^[^ ^21^ ^]^ coconut shells,^[^ ^22^ ^]^ bamboo stalks,^[^ ^23^ ^]^ lotus leaves,^[^ ^24^ ^]^ and rice husks.^[^ ^25^ ^]^ The primary factors influencing biomass selection are its cost‐effectiveness and regional availability.^[^ ^20^ ^]^

Among these, date palm biomass is particularly attractive due to its abundance, low cost, and favorable composition. Date palm trees, widely cultivated in the Middle East and North Africa, generate substantial amounts of solid biomass waste, including date stones. On average, each date palm tree produces ≈12.5 kg of date stone biomass waste annually. Date stones contain balanced fractions of cellulose, hemicellulose, and lignin, making them an ideal precursor for carbon material synthesis.^[^ ^26^ ^]^ However, there is a significant lack of studies investigating date stone biomass as a precursor for supercapacitor electrodes, and achieving desirable properties necessitates physical and chemical alteration. Potassium carbonate (K_2_CO_3_) and melamine (C_3_H_6_N_6_) are individually recognized as effective activating agents and nitrogen dopants, respectively.^[^ ^27^, ^28^ ^]^ However, their combined effects on the structural and electrochemical properties of activated carbon remain largely unexplored. To bridge this gap, the present study investigates the feasibility of utilizing date stone biomass as a renewable source for developing high‐performance supercapacitor electrode materials, specifically examining the synergistic influence of K_2_CO_3_ activation and melamine doping on its microstructural evolution, porosity, and electrochemical performance.

## 2 Result and Discussion

Date stone‐derived activated carbon was prepared using a two‐stage pyrolysis process that included melamine (C_3_H_6_N_6_) as a nitrogen dopant and potassium carbonate (K_2_CO_3_) as an activating agent. X‐ray diffraction (XRD) analysis revealed the microstructural evolution of activated carbon materials, with two characteristic peaks at ≈22°–23° and 42°–43° corresponding to the (002) and (100) crystal planes, respectively. The addition of K_2_CO_3_ induced a shift in the (002) peak toward lower angles, suggesting an increase in the interplanar spacing. Indeed, the C:K:M sample exhibited the largest interplanar spacing (*d*_002_ = 4.3 Å), followed by C:K (3.95 Å), C (3.78 Å), and C:M (3.62 Å). Typically, biomass‐derived activated carbons exhibit larger interplanar spacings than pure graphite (3.35 Å) owing to their turbostratic nature.^[^ ^29^ ^]^ Both the biomass source and the processing conditions employed influence this characteristic, with higher processing temperatures leading to a decrease in the interplanar spacing.^[^ ^30^ ^]^

The incorporation of melamine resulted in a decrease in the average crystallite height (Lc), while K_2_CO_3_ activation promoted crystallite height growth, as reflected in the Lc values of 0.30, 0.42, 0.26, and 0.33 Å for C, C:K, C:M, and C:K:M, respectively. Conversely, K_2_CO_3_ addition alone resulted in a decrease in the average crystallite diameter (La) of the C:K sample (0.08 Å), while the C, C:M, and C:K:M samples exhibited relatively similar diameters (≈0.1 Å). This observation slightly deviates from previously reported findings on date stone‐derived carbon, likely due to variations in biomass source and specific processing conditions.^[^ ^31^ ^]^

**Figure 1:**
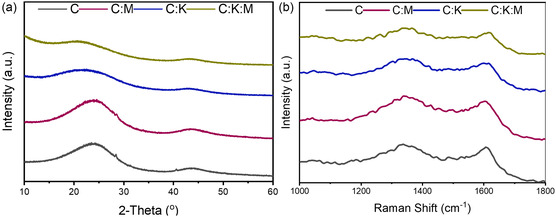
a) XRD patterns and b) Raman spectra of C, C:K, C:M, and C:K:M samples.

The incorporation of K_2_CO_3_ resulted in reduced peak intensity and broadening of the (002) and (100) diffraction peaks, suggesting the formation of highly defective carbon materials. To further investigate the degree of graphitization, Raman spectroscopy was conducted. The ratio of the *D* band (≈1345 cm^−1^) to the *G* band (≈1605 cm^−1^), denoted as *I*_G_/*I*_D_,^[^ ^32^ ^]^ was calculated (**Figure** **1**b). The pure carbon sample exhibited the highest degree of graphitization, with an *I*_G_/*I*_D_ ratio of 0.97. However, the addition of either melamine or K_2_CO_3_ led to a slight increase in defect concentration, as evidenced by smaller *I*_G_/*I*_D_ values (0.95) for both samples. Regardless, none of the prepared carbons displayed an *I*_G_/*I*_D_ ratio of >1, confirming the defective nature of all the materials. It is important to note that all the samples in this study were processed under identical thermal conditions, although the *I*_G_/*I*_D_ ratio is primarily influenced by temperature.^[^ ^32^, ^33^ ^]^

**Figure 2:**
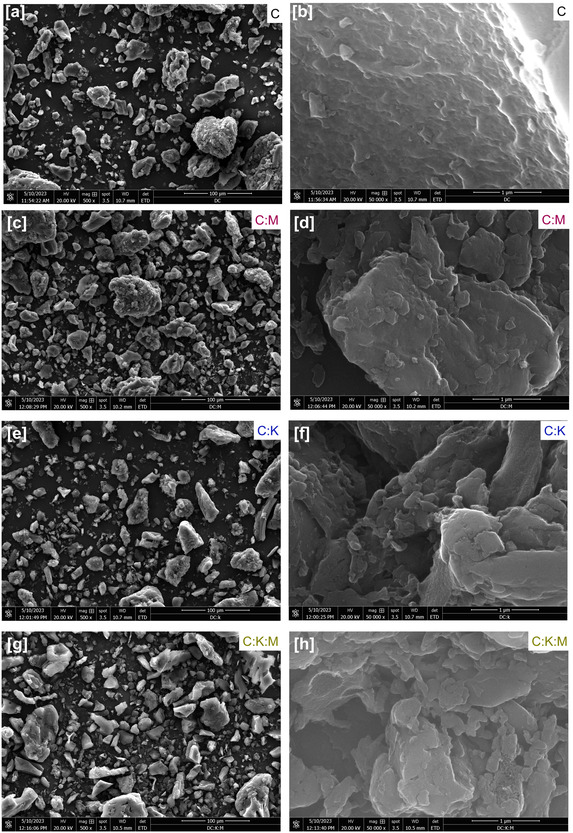
SEM images at different magnifications and energy‐dispersive X‐ray spectroscopy analysis of a,b) C, c,d) C:M, e,f) C:K, and g,h) C:K:M sample.

The microstructural features of the synthesized carbon materials were examined using scanning electron microscopy (SEM). The SEM images (**Figure** **2**) of C (2a, 2b), C:M (2c, 2d), C:K (2e, 2f), and C:K:M (2g, 2h) revealed a heterogeneous distribution of particle sizes with irregular morphologies. Notably, the C:K and C:K:M samples exhibited a tendency toward smaller particle sizes than the C and C:M samples, suggesting a potential increase in the specific surface area.

**Figure 3:**
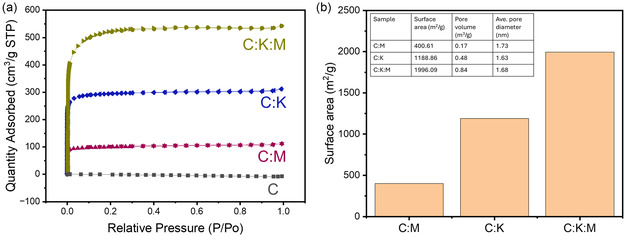
a) Nitrogen adsorption–desorption isotherms of the C, C:M, C:K, and C:K:M samples. b) Summary of Brunauer–Emmett–Teller (BET) surface area, pore volume, and average pore diameter of each sample.

The nitrogen adsorption–desorption isotherms (**Figure** **3**a,b) for all samples (C:M, C:K, and C:K:M) exhibit Type I behavior according to the IUPAC classification. This type of isotherm is typical of microporous materials, with pore sizes predominantly in the super‐microporous range (<2 nm). The sharp rise in nitrogen uptake at low relative pressures at the start showed that the micropores had a strong attraction for nitrogen.

The addition of either melamine or K_2_CO_3_ significantly enhanced the surface area and pore volume of the carbon materials. C:M exhibits a surface area of 400.16 m^2^ g^−1^ and a pore volume of 0.17 cm^3^ g^−1^, which is higher than that of pure carbon, where a minimal surface area was generated. The C:K sample shows a substantial increase to 1188.86 m^2^ g^−1^ and 0.47 cm^3^ g^−1^, respectively. The combined effect of melamine and K_2_CO_3_ in the C:K:M sample further elevates these values to 1996.09 m^2^ g^−1^ and 0.84 cm^3^ g^−1^, respectively. The K_2_CO_3_ addition and its enhancement have been well documented^[^ ^34^, ^35^ ^]^ and the effect of melamine on enhancing the surface area has been reported;^[^ ^36^ ^]^ however, synergetic melamine addition with an activating agent has been rarely reported.^[^ ^37^ ^]^ Interestingly, despite the huge increase in surface area and pore volume, the average pore diameter remained relatively stable across all samples, suggesting the generation of new micropores rather than a significant widening of existing ones. The C:K:M sample is expected to perform the best as a supercapacitor because it has the greatest pore volume and surface area among the prepared carbon samples. In addition to its impact on physical properties, the potential of melamine in promoting nitrogen incorporation is an area that requires further examination.

**Figure 4:**
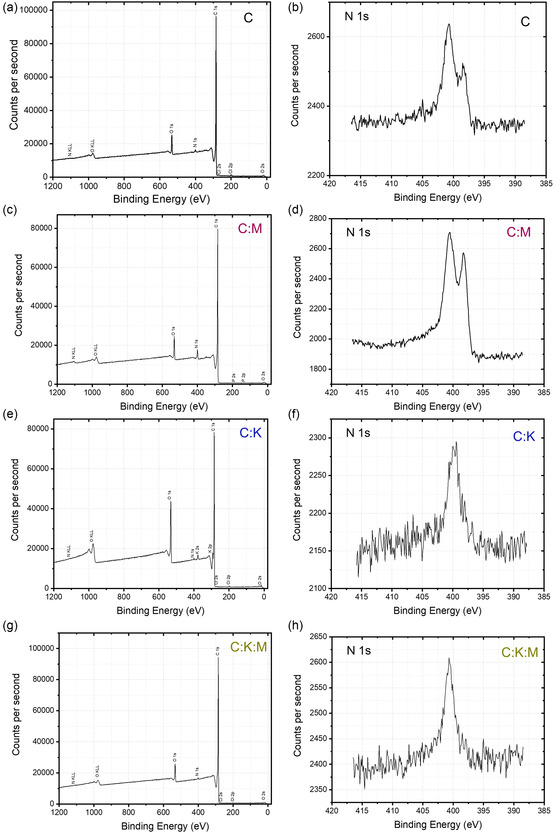
XPS analysis of C, C:M, C:K, and C:K:M samples. a–d) Survey spectra showing the elemental composition of the samples. e–h) High‐resolution N1s spectra revealing the different nitrogen species present in each sample.

In addition to their surface area and pore volume, the supercapacitor efficacy of carbon‐based electrodes is significantly improved by the presence of heteroatoms, such as nitrogen and oxygen. This is due to the enhancement of conductivity and wettability properties. Therefore, we conducted a more thorough analysis of the elemental composition and chemical bonding, we determined the effects of K_2_CO_3_ and melamine using X‐ray photoelectron spectroscopy (XPS). Initially, the expected predominance of C1s and O1s peaks is confirmed by survey scans (**Figure** **4**a–d) of the C, C:M, C:K, and C:K:M samples. The smaller N1s peaks are ascribed to both the residual nitrogen inherently present in the biomass precursor and melamine addition.

The high‐resolution N1s spectra (Figure 4e–h) reveal distinct peaks at ≈398 eV and 400 eV for the C and C:M samples, which correspond to pyridinic and pyrrolic nitrogen species, respectively. This shows that the pure date stone already has some nitrogen content.^[^ ^9^ ^]^ In contrast, the C:K and C:K:M samples exhibit higher noise levels and a peak centered around 400 eV, indicative of a predominance of pyrrolic nitrogen and a lower overall nitrogen concentration. This suggests that the activating agent may play a role in minimizing pyridinic nitrogen formation.

**Figure 5:**
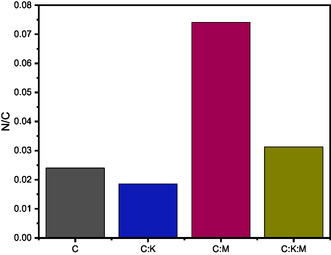
N/C atomic ratio determined by elemental analysis.

Elemental analysis was performed (**Figure** **5**) for the rigid quantification of the nitrogen content. The N/C ratio was the highest for C:M (0.074), followed by C:K:M (0.031), C (0.024), and C:K (0.018), confirming the successful incorporation of nitrogen with the addition of melamine to the carbon materials. In addition, this confirms that the incorporation of K_2_CO_3_ has a leaching effect, as evidenced by the lower nitrogen concentration of C:K:M (0.31) compared to C:M (0.074) and C:K (0.018) compared to pure C (0.024).

Next, the electrochemical performance of the synthesized date stone‐derived activated carbon was systematically evaluated to investigate the effects of K_2_CO_3_ activation and melamine doping. The C:M, C:K, and C:K:M samples were analyzed using cyclic voltammetry (CV) and galvanostatic charge–discharge (GCD) measurements to assess their capacitive behavior and energy storage capabilities.

**Figure 6:**
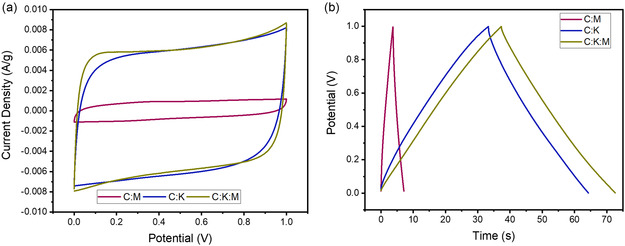
a) CV curves at a scan rate of 50 mV s^−1^. b) GCD profiles at a current density of 5 A g^−1^ for C:M, C:K, and C:k:M samples.

The CV curves (**Figure** **6**a) clearly demonstrate that the areas under the curves for the C:K and C:K:M samples are significantly larger than that of the C:M sample, indicating an improvement in charge storage capacity upon K_2_CO_3_ activation. This suggests that chemical activation enhances the electrochemical properties of date stone‐derived carbon by increasing accessible surface area and porosity. Furthermore, the C:K:M sample exhibits a slightly larger CV area than the C:K sample, suggesting a further enhancement in charge storage due to nitrogen incorporation and an enlarged surface area facilitated by melamine doping.

To further quantify the capacitance and energy storage properties, GCD measurements were performed (Figure 6b). The C:M sample exhibits a specific capacitance of 17.25 F g^−1^, resulting in energy and power densities of 2.4 Wh kg^−1^ and 2500 W kg^−1^, respectively. Although melamine doping increases surface area, it does not significantly enhance electrochemical performance to a desirable level, indicating that surface area alone is insufficient for high‐capacity charge storage.

In contrast, the C:K and C:K:M samples exhibit substantially higher specific capacitances, measuring 155.65 and 176.2 F g^−1^ at 5 A g^−1^, respectively. These values correspond to energy densities of 21.26 and 24.47 Wh kg^−1^, when operating at a power density of 2500 W kg^−1^. The addition of K_2_CO_3_ as an activating agent significantly improves the electrochemical performance by enhancing porosity and conductivity. Additionally, the further incorporation of melamine leads to a 13.2% increase in capacitance, highlighting the synergistic role of surface area enhancement and nitrogen doping in improving charge storage efficiency.

**Figure 7:**
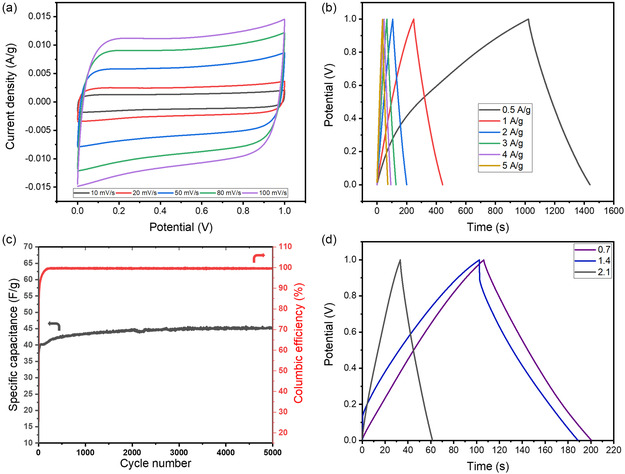
a) CV curves at different scan rates (10–100 mV s^−1^). b) GCD profiles at various current densities (0.5–5 A g^−1^). c) Cycling stability and Coulombic efficiency of the C:K:M sample at a current density of 10 A g^−1^ for 5000 cycles of C:K:M samples. d) GCD profiles at various mass loading (0.7, 1.4, and 2.1 mg).

For the optimal C:K:M sample, CV curves recorded at various scan rates (**Figure** **7**a) demonstrate that the curve area increases as the scan rate increases. This phenomenon is attributed to the limited time available for ion diffusion into the electrode's porous network at higher scan rates. Consequently, ions primarily accumulate at the outer surface of the electrode, leading to a larger capacitive current and a more rectangular CV shape, characteristic of electric double‐layer capacitive behavior.

To further evaluate the rate capability, GCD measurements were conducted at different current densities (Figure 7b). As expected, higher current densities resulted in shorter discharge times, indicating a reduction in capacitance. This behavior arises due to increased resistance and limited ion diffusion kinetics at higher current densities, which restrict the full utilization of the electrode's available surface area.

The C:K:M sample exhibited specific capacitances of 209.36, 198.17, 190.48, 182.46, 179.12, and 176.2 F g^−1^ at current densities of 0.5, 1, 2, 3, 4, and 5 A g^−1^, respectively. These capacitance values correspond to energy densities of 29.08, 27.5, 26.46, 25.34, 24.88, and 24.47 Wh kg^−1^ at power densities of 250, 500, 1000, 1500, 2000, and 2500 W kg^−1^, respectively. The C:K:M composite exhibits high rate capability, maintaining high capacitance and energy density with minimal losses at elevated current densities, underscoring its efficient charge storage mechanism.

The cycling stability of the C:K:M sample was further examined by subjecting it to 5000 charge–discharge cycles at 10 A g^−1^ (Figure 7c). The capacitance retention remained excellent, with no apparent signs of degradation. Notably, during the initial charge–discharge cycles, a gradual increase in capacitance was observed, which can be attributed to an activation process wherein the electrolyte progressively penetrates the pores, thereby expanding the accessible surface area for charge storage. This phenomenon has been widely reported in carbon‐based supercapacitors and is commonly associated with the gradual wetting and activation of micropores over successive cycles, further enhancing the electrochemical performance of the material.

To further evaluate the practical applicability of the synthesized materials, the active material mass loading was varied from 0.7 to 1.4 mg and 2.1 mg, and the corresponding specific capacitances were determined (Figure 7d). The values were 190.48, 171.95, and 56.56 F g^−1^, respectively, showing a decline in capacitance with increasing mass loading due to limited electrolyte accessibility and ion diffusion constraints, which reduce the effective utilization of the active material. To provide a more comprehensive assessment, the absolute capacitance was calculated based on total charge storage capacity, yielding values of 0.133, 0.241, and 0.119 F for mass loadings of 0.7, 1.4, and 2.1 mg, respectively. Notably, the 1.4 mg electrode exhibited the highest absolute capacitance, suggesting an optimal balance between mass loading and electrochemical accessibility. In contrast, the 2.1 mg electrode showed a significant drop in capacitance, reinforcing the diffusion limitations. Finally, a comparative **Table** **1** has been incorporated to benchmark the electrochemical performance of the synthesized material against previously reported studies, providing a broader context for its applicability and competitiveness.

**Table 1:** Comparison of electrochemical performance of biomass‐derived supercapacitors.

Material	Electrolyte	Specific Capacitance [F g^−1^]	Current Density [A g^−1^]	Capacitance retention [%] cycle‐index	References
Laurus nobilis leaves	1 m H_2_SO_4_	205	1	–	[38]
Chitosan aerogel	1 m H_2_SO_4_	168	0.5	–	[39]
camellia	1 m KOH	125.42	2	92.51% 4,000 cycles 5 A g^−1^	[40]
Starch	6 m KOH	49.77	1	90% 20,000 cycles 10 A g^−1^	[41]
Palm kernel shell	1 m KOH	210	0.5	95–97% after 1000 cycles 5 A g^−1^	[42]
Pinecone	1 m H_2_SO_4_	185	0.5	96.1% after 10,000 cycles 5 A g^−1^	[43]
Nut‐skin waste	1 m H_2_SO_4_	193	0.5	97% after 10,000 cycles 5 A g^−1^	[44]
Date stone	1 m H_2_SO_4_	209.36	0.5	120% 5,000 cycles 10 A g^−1^	This work

## 3 Conclusion

In conclusion, this study successfully demonstrated the synergistic effect of K_2_CO_3_ activation and melamine doping on the development of high‐performance supercapacitor electrodes derived from date stone biomass. The C:K:M composite exhibited a substantially increased surface area and pore volume compared to the C:M and C:K samples, along with a higher nitrogen content than the C:K sample, emphasizing the enhanced porosity and nitrogen incorporation achieved through combined activation and doping strategies.

The unique combination of these physicochemical properties resulted in exceptional electrochemical performance, with a specific capacitance of 209.36 F g^−1^ at 0.5 A g^−1^, cycling stability exceeding 5000 cycles at a high current density of 10 A g^−1^, and high energy and power densities of 24.47 Wh kg^−1^ at 2500 W kg^−1^. These results underscore the potential of date stone‐derived carbon as a sustainable and cost‐effective precursor for next‐generation energy storage materials.

## 4 Experimental Section

4.1

4.1.1

##### Preparation of Date Stone‐Derived Activated Carbon

First, the date stones were thoroughly washed multiple times with distilled water to remove impurities. The cleaned samples were then dried in an oven at 110 °C for 24 h. Once dried, the date stones were ground into fine powder using a high‐speed blender and subsequently carbonized at 400 °C in a tube furnace. The heating and cooling rates were maintained at 10 and 5 °C min^−1^, respectively, under a continuous flow of nitrogen gas to ensure an inert atmosphere.

To investigate the influence of K_2_CO_3_ activation and melamine doping, four different sample compositions were prepared by physically mixing specific ratios of materials. 1) Pure date stone‐derived carbon (C); 2) carbon–melamine mixture (C:M, 1:0.6 ratio); 3) carbon–K_2_CO_3_ mixture (C:K, 1:4 ratio); and 4) carbon–K_2_CO_3_‐melamine mixture (C:K:M, 1:4:0.6 ratio). Following this, all samples were heated to 850 °C in a nitrogen (N_2_) atmosphere and then cooled to room temperature, with heating and cooling rates maintained at 10 and 5 °C min^−1^, respectively.

##### Fabrication of Date Stone‐Derived Activated Carbon Electrodes

The prepared activated carbon, carbon black, and polyvinylidene fluoride binder were thoroughly mixed using a mortar and pestle in a 7.5:1.5:1 weight ratio, respectively, for ≈20 min until a homogeneous mixture was obtained. The resulting homogeneous mixture was dissolved in an appropriate amount of N‐methyl‐2‐pyrrolidone and stirred continuously at 90 °C for ≈3 h to form a uniform slurry. This slurry was then drop cast onto a stainless‐steel foil, ensuring even distribution of the active material. Each electrode contained ≈0.7 mg of the active material unless otherwise specified.

##### Characterization

The crystallographic structures of the prepared carbon materials were analyzed using XRD with a Rigaku MiniFlex diffractometer, utilizing Cu Kα radiation (*λ* = 1.5418 Å). In addition, the crystallite size parameters, including La (crystallite diameter), Lc (crystallite height), and *d*_0__0__2_ (interplanar spacing), were calculated using XRD. The interplanar spacing ($d_{002}$) was determined using Bragg's equation $d_{002}=\frac{\lambda }{2\sin\theta }$, where *λ* represents the X‐ray wavelength and *θ* is the diffraction angle. The crystallite height (Lc) and the crystallite diameter (La) were calculated using the Scherrer equation $L_{c/a}=\left(\frac{K\lambda }{\beta \cos\theta }\right)$. The (002) peak center was utilized to determine the crystallite height (Lc), with a shape factor (*K*) of 0.89. Similarly, the (100) peak data were employed to calculate the crystallite diameter (La), using a shape factor of 1.84. The parameter *β* represented the full width at half maximum of the respective diffraction peak.^[^ ^20^ ^]^

In addition, the surface area and porosity of the samples were determined using nitrogen adsorption–desorption isotherms at 77 K, measured with a Micromeritics 3Flex surface characterization analyzer. Furthermore, the chemical composition and bonding states of the carbon materials were examined via XPS , conducted on a Kratos AXIS Supra spectrometer.

##### Electrochemical Measurements

The electrochemical performance of the prepared date stone‐derived activated carbon was evaluated in a symmetric supercapacitor device using the CHI 760E electrochemical workstation. A filter paper separator was used, and activated carbon served as both the anode and cathode. Electrochemical measurements were conducted using 1 m H_2_SO_4_ electrolyte within a voltage window of 0–1.0 V. Different scan rates, ranging from 10 to 150 mV s^−1^, were used for the CV measurements. Different current densities ranging from 0.5–5 A g^−1^ were used for the GCD approach. The stability of the manufactured supercapacitor was tested at 10 A g^−1^ for 5000 cycles. The specific capacitance (*C*), energy density (*E*), and power density (*P*) were calculated using the following equations.

(1) $$C=\frac{I\Delta V}{m\Delta t}$$

(2) $$E=\frac{1}{2}C{\left(\Delta V\right)}^{2}$$

(3) $$P=\frac{E}{\Delta t}$$

where *I* is the discharge current (A), Δt is the discharge time (s), *m* is the mass of active material (g), and ΔV is the potential window (V).

## Conflict of Interest

The author declares no conflict of interest.

## Data Availability

The data that support the findings of this study are available from the corresponding author upon reasonable request.
